# Self‐Assembly of Lamellae‐in‐Lamellae by Double‐Tail Cationic Surfactants

**DOI:** 10.1002/advs.202401210

**Published:** 2024-05-15

**Authors:** Zhixuan Zhong, Guanqun Du, Linbo Ma, Yilin Wang, Jian Jiang

**Affiliations:** ^1^ Beijing National Laboratory for Molecular Sciences State Key Laboratory of Polymer Physics and Chemistry Institute of Chemistry Chinese Academy of Sciences Beijing 100190 P. R. China; ^2^ University of Chinese Academy of Sciences Beijing 100049 P. R. China; ^3^ CAS Key Laboratory of Colloid Interface and Chemical Thermodynamics CAS Research/Education Center for Excellence in Molecular Sciences Beijing National Laboratory for Molecular Science Institute of Chemistry Chinese Academy of Sciences Beijing 100190 P. R. China

**Keywords:** double‐tail surfactant, hierarchical self‐assembly, lamellae‐in‐lamellae, molecular dynamics simulation, SAXS

## Abstract

The molecular structures of surfactants play a pivotal role in influencing their self‐assembly behaviors. In this work, using simulations and experiments, an unconventional hierarchically layered structure in the didodecyldimethylammonium bromide (DDAB)/water binary system: lamellae‐in‐lamellae is revealed, a new self‐assembly structure in surfactant system. This self‐assembly structure refers to a lamellar structure with a shorter periodic length (inner lamellae) embedded in a lamellar phase with a longer periodic length (outer lamellae). The normal vectors of these two lamellar regions orient perpendicularly. In addition, it is observed that this lamellar‐in‐lamellar phase disappears when the two tails of the cationic surfactants become longer. The formation of the lamellar‐in‐lamellar architecture arises from multiple interacting factors. The key element is that the short tails of the DDAB surfactants enhance hydrophilicity and rigidity, which facilitates the formation of the inner lamellae. Moreover, the lateral monolayer of the inner lamellae provides shielding from the water and prompts the formation of the outer lamellae. These findings indicate that molecular structures and flexibility can profoundly redirect the hierarchical self‐assembly behaviors in amphiphilic systems. More broadly, this work presents a new strategy to deliberately program hierarchical nanomaterials by designing specific surfactant molecules to act as tunable scaffolds, reactors, and carriers.

## Graphical abstract

An unconventional hierarchically layered structure: lamellae‐in‐lamellae, is discovered in the double‐tail surfactant/water system through molecular dynamics simulations and experiments. The lamellae‐in‐lamellae is assembled by a lamellar structure with a shorter periodic length (inner lamellae) embedded perpendicularly in a lamellar phase with a longer periodic length (outer lamellae).

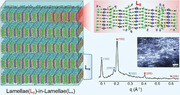

## 1 Introduction

Hierarchy is ubiquitous across natural and engineered systems, spanning multiple organizational levels from nanoscale to macroscale. This multi‐scale ordering, where components at each level interact to form ordered structures, provides systems with emergent collective properties beyond each individual constituent.^[^ ^1^, ^2^, ^3^ ^]^ Hierarchical structures are crucial in proteins in which secondary peptide structures encode 3D folds to enable complex bioactivities.^[^ ^4^ ^]^ Cellular organization gives rise to tissue hierarchy and functional organs.^[^ ^5^, ^6^ ^]^ Likewise, engineered superstructures gain unique mechanical and optical properties from hierarchies between designed micro/nanostructures.^[^ ^7^, ^8^, ^9^, ^10^ ^]^ Therefore, this multi‐scale ordering underpins the utility of hierarchical fabrication for constructing functional materials.

Surfactant systems universally exhibit hierarchical architectures, which influence a wide range of natural phenomena and industrial applications. Their specific structures and interactions determine this multi‐scale organization. With hydrophilic headgroups and hydrophobic tails, surfactant molecules can readily form bilayer structures which serve as the basis for many self‐assembly systems including vesicles, tubular structures, and bicontinuous phases.^[^ ^11^, ^12^, ^13^ ^]^ These organized assemblies can further construct complex hierarchical architectures or aggregates such as hydrogels of lamellar phases,^[^ ^14^ ^]^ vesicle networks crosslinked by dynamic imine‐based covalent bonds,^[^ ^15^ ^]^ biomimetic myelin figures based on tubular phospholipid bilayers,^[^ ^16^ ^]^ and chiral mesoporous nanospheres from the helical assembly of disk‐like micelles.^[^ ^17^ ^]^ These examples demonstrate that precise control over surfactant molecular interactions at varying length scales allows the fabrication of composite materials with diverse customized morphologies and functionalities. Hierarchical complexity arises from competing intermolecular and intramolecular interactions including hydrophobic, electrostatic, hydrogen bonding, and steric factors.^[^ ^18^, ^19^, ^20^ ^]^ Therefore, further control over self‐assembly behaviors could be achieved by modulating molecular configuration and flexibility.

In this work, we employed coarse‐grained molecular dynamics simulations (CGMD) and experimental methods of small angle X‐ray scattering (SAXS) and polarized optical microscopy (POM) to unveil a distinctive hierarchical architecture within the double‐tail cationic surfactant/water binary systems, referred to as “lamellae‐in‐lamellae”. The presence of this hierarchically layered structure not only offers a new perspective in understanding the self‐assembly mechanisms of surfactant systems but also holds important implications for the design of novel materials with specific functionalities.

## 2 Results and Discussion

**Figure 1:**
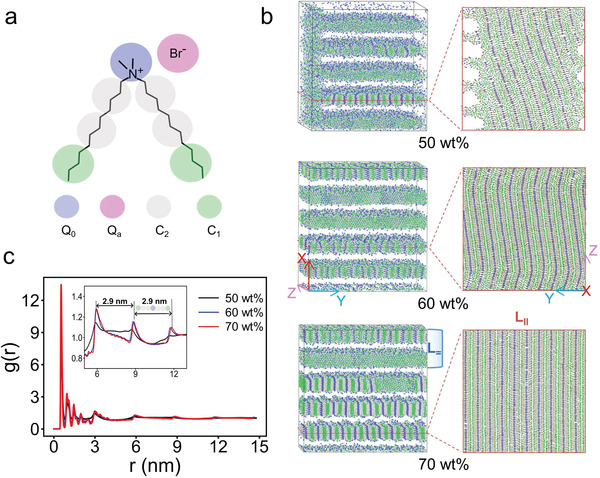
a) Coarse‐grained mapping of DDAB molecule; details about the bead type of *Q*_0_, *Q*_*a*_, *C*_2_, and *C*_1_ can be found in Computational Section; b) snapshots and slices of 50, 60, and 70 wt% DDAB/water binary systems, water and bromine ions (Br^−^) are visually hidden for clarity; c) radial distribution functions between headgroups of DDAB.

As a highly effective tool for investigating microscopic systems, coarse‐grained molecular dynamics simulations can be conducted to elucidate hierarchical structures in surfactant/water mixtures. Here, we utilized the Martini model to construct a coarse‐grained mapping of the didodecyldimethylammonium bromide (DDAB) molecule (**Figure** **1**a).^[^ ^21^, ^22^ ^]^ To validate our model, the evaluation is performed using the critical micelle concentration (CMC) as a key metric compared with the experiment values.^[^ ^23^, ^24^ ^]^ As shown in Figure S1 (Supporting Information), utilizing the standard Martini water model (SW) with Particle Mesh Ewald algorithm (PME), the results not only exhibit an agreement with experimental CMC values but also demonstrate robust stability across varying box sizes. In this work, DDAB/water binary systems were systematically investigated across a broader range of mass concentrations (1–80 wt%). Strikingly, the emergence of lamellae‐in‐lamellae, a novel self‐assembly structure in double‐tail surfactant systems, is detected within the DDAB mass concentration range spanning from 50 to 70 wt%. Therefore, our analysis was directed toward three distinct systems characterized by varying surfactant mass concentrations: 50, 60, and 70 wt%. (For more detailed results concerning dilute examples, including concentrations of 1, 5, and 20 wt%, please refer to Figure S2, Supporting Information). Each system was carried out over a duration of 4 µs with three independent replicas, enabling us to gain valuable and robust insights into the self‐assembly behaviors of these intricate mixtures. The equilibrium state within the simulation time is characterized by the density and potential energy of the system in Figure S3 (Supporting Information).

As clearly shown in Figure 1b and Figure S4a–d (Supporting Information), it is occurred that the lamellar phases in the DDAB/water binary systems are formed under three different concentrations. Moreover, a hierarchical structure assembled by DDAB molecules is observed. This hierarchical structure is a lamellar‐in‐lamellar structure which refers to a lamellar phase with a shorter periodic length (inner lamellae, denoted as *L*_∥_) embedded in a lamellar phase with a longer periodic length (outer lamellae, denoted as *L*_═_). The normal vectors of these two lamellar regions orient perpendicularly. In Figure 1c, the radial distribution function (RDF) profiles between the headgroups show a series of long‐range ordered peaks spanning from 6.0 to 12.0 nm, indicating an ordered arrangement of DDAB molecules in these systems. The distance between adjacent peaks is estimated to be 2.90 nm, which closely corresponds to the length of the DDAB molecule with two tails fully stretched to two opposite sides within the inner lamellar structures (*L*_∥_).

**Figure 2:**
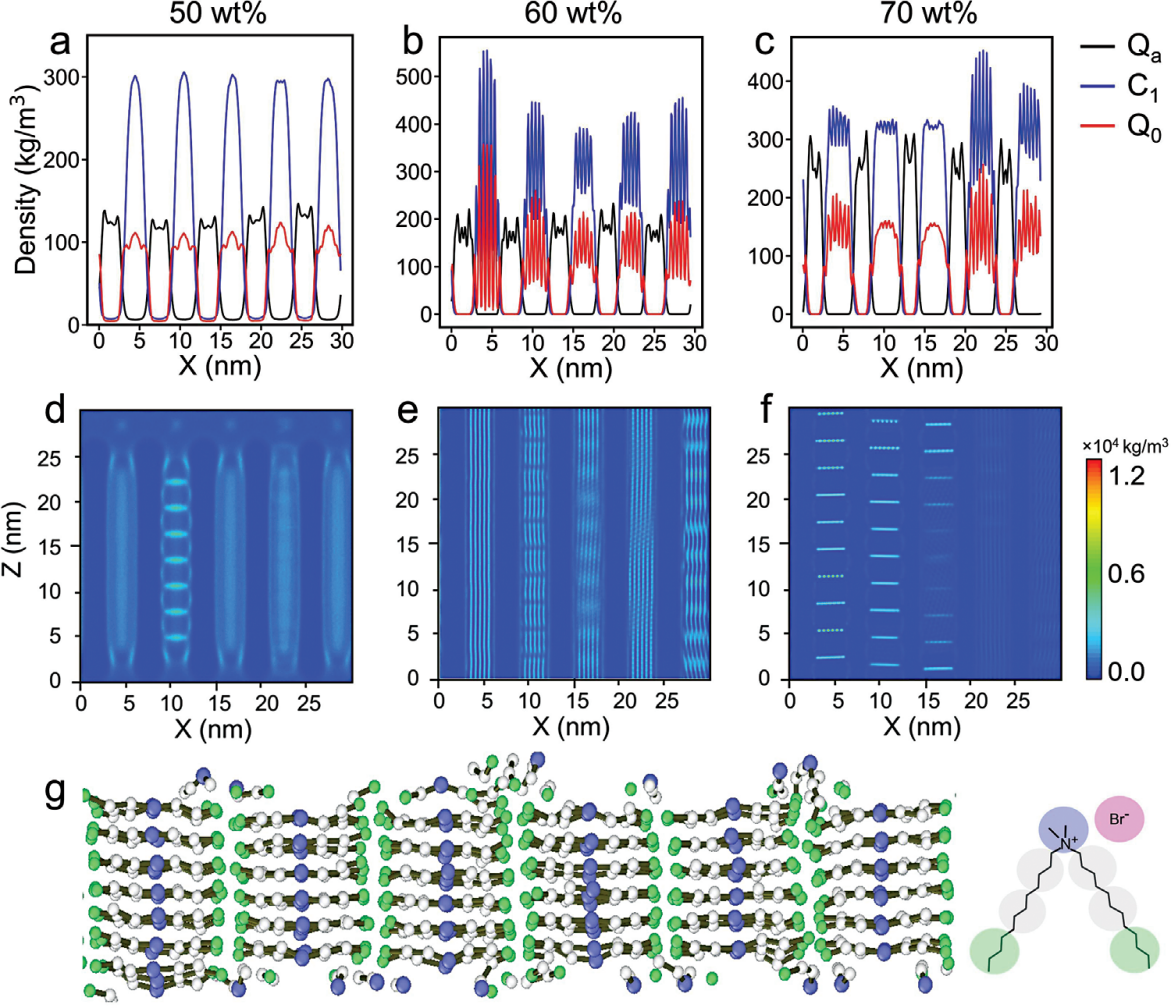
1D density distribution of a) 50 wt%, b) 60 wt%, and c) 70 wt% DDAB/water binary systems, *Q*_*a*_ is the bromine ion bead, *C*_1_ is the terminal bead of each tail of DDAB, and *Q*_0_ is the headgroup bead of DDAB; 2D density maps of headgroup of d) 50 wt%, e) 60 wt%, and f) 70 wt% DDAB/water binary systems; g) slice snapshot of inner lamellar structures (*L*_∥_), and the representation of DDAB molecule.

To further delineate the lamellar‐in‐lamellar structure, the 1D density profiles of each component and 2D density maps of the headgroup are plotted in **Figure** **2**. In the case of 50 wt% DDAB/water system, the periodic occurrence of terminal bead in the tail (*C*_1_) and headgroup (*Q*_0_) peaks distinctly signifies a perpendicular lamellar‐in‐lamellar motif (Figure 2a). These density profiles align consistently with existing findings about the lamellar‐in‐lamellar structure of block copolymer systems in the literature.^[^ ^25^, ^26^ ^]^ As the DDAB concentration increases to 60 and 70 wt%, the *L*_∥_ lamellae profiles (Figure 2b,c) exhibit more pronounced and well‐organized characteristics, indicating a heightened prominence and organization to the hierarchical structure. Two distinct patterns of lamellar sub‐structures (*L*_∥_) emerge: the ripple arrangements and parallel stacked arrays in Figure 1b, which can be attributed to the effects of periodic boundary conditions (PBC). In both cases, DDAB molecules pack into the layers with a large angle (≈180°) between the two stretched tails, as exemplified in Figure 2g and Figure S4e (Supporting Information). Additionally, the headgroup peaks and patterns of 2D density maps in Figure 2 manifest at the layer–water interface, analogous to the characteristic features of the bilayer membrane. Similar phenomena are also observed in another two independent replicas in Figures S5–S8 (Supporting Information).

**Figure 3:**
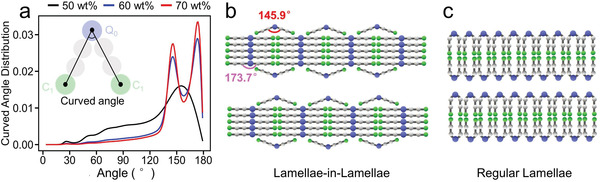
a) The curved angle distributions of DDAB; the illustration of b) lamellae‐in‐lamellae and c) regular lamellae.

To deeply clarify the molecular arrangement at the DDAB layer‐water interface, the in situ curved angle of DDAB molecules is calculated from CGMD trajectories. Here, the curved angle refers to the angle between the two vectors connecting the headgroups bead (*Q*_0_) and the terminal bead (*C*_1_) of each two tails as depicted in the inset of **Figure** **3**a.^[^ ^27^ ^]^ In Figure 3a, for the 60 and 70 wt% cases, two distinctive peaks appear at 145.9° and 173.7°, while the 50 wt% case only exhibits one broader peak at ≈150.0°. The peak at 145.9° in the 60 and 70 wt% cases corresponds to the curved angles of the bent DDAB molecules in the outermost monolayer of the inner *L*_∥_ lamellae, while the peak at 173.7° represents the hydrophobic alkyl chains of DDAB in a linear, fully‐stretched arrangement in the inner *L*_∥_ lamellae (Figure 3b). This reinforces our above analysis of density maps and demonstrates that the dominance of the hierarchical *L*_∥_ structure increases in proportion with the rise of DDAB concentration in the binary system. Such a huge curved angle implies that this hierarchically lamellar structure deviates from a normal bilayer observed in our previous work.^[^ ^27^ ^]^ Additionally, the average curved angle (**Table** **1**) increases with concentration, revealing that it is more readily to form lamellar‐in‐lamellar structures at higher DDAB concentrations. Notably, counterions are more likely concentrated in the water phases and are not proximal to headgroups, thereby, forming periodical condensed counterion layers, which makes the lamellar structure more stable.^[^ ^28^, ^29^ ^]^ This phenomenon, while appearing to violate local charge neutrality, is actually a result of the unique spatial constraints for counterions and the balance of various interactions within our system. Based on the comprehensive analysis presented above, it is evident that the hierarchical layers formed in DDAB/water binary systems are distinctly characterized as lamellar‐in‐lamellar structures. As illustrated in Figure 3b, the structure consists of stacked lamellae (*L*_═_) with smaller perpendicular lamellar domains (*L*_∥_) periodically arranged. And each inner lamellae is enveloped by two DDAB monolayers to prevent it from exposure to the aqueous environment. Although part of the tails in the monolayers are exposed to the water, the system has attained a state that is energetically favorable.

**Table 1:** Average curved angle and filled density of DDAB/water binary systems.

wt%	Average Curved Angle [°]	Average Filled Density [kg m^−3^]
50	125.66 ± 0.36	1247.98 ± 2.07
60	146.88 ± 0.19	1319.27 ± 1.55
70	152.48 ± 0.18	1344.54 ± 1.16

**Figure 4:**
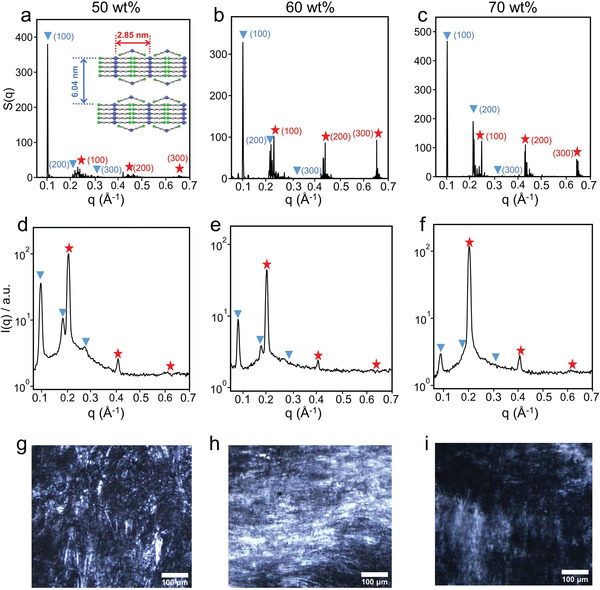
a–c) Structure factor, d–f) SAXS curves, and g–i) POM images of DDAB/water binary systems at DDAB concentrations of (a, d, g) 50 wt%, (b, e, h) 60 wt%, and (c, f, i) 70 wt%. The Bragg scattering peaks for the outer lamellae (*L*_═_) and inner lamellae (*L*_∥_) in (a–f) are marked with blue triangles and red stars, respectively.

For the purpose of validating the lamellar‐in‐lamellar structure experimentally, small angle X‐ray scattering (SAXS) measurements were performed and the results are compared with the structure factor (S(q)) calculated from the simulations. In the case of 50 wt% DDAB system, the S(q) profile (**Figure** **4**a) shows characteristic peaks at *q* = 0.104 Å^−1^, 0.210 Å^−1^, and 0.313 Å^−1^ labeled with triangles with the ratio of 1:2:3 typically for lamellar liquid crystal phase, the periodic length (*d*) of the outer lamellae (*L*_═_) can be estimated from the first‐order peak, i.e., *d* = 2π/*q* = 6.04 nm. In addition, it is obvious that the peaks (marked with stars) for a well‐ordered inner lamellae (*L*_∥_) can also be found in Figure 4a (the first‐order peak appears at *q* = 0.220 Å^−1^), and the periodic length of the inner lamellae (*L*_∥_) is *d* = 2π/*q* = 2.85 nm, which is in good agreement with the distance (2.90 nm) between the adjacent peaks revealed in the RDF profiles (Figure 1c). Meanwhile, the lamellar‐in‐lamellar structure formed in DDAB/water binary systems is also evidenced by SAXS measurements. As shown in Figure 4d, the Bragg scattering peaks with the ratio of 1:2:3 for the outer lamellae (*L*_═_) and inner lamellae (*L*_∥_) in 50 wt% DDAB system can be clearly distinguished. Moreover, the first‐order Bragg peaks appear at *q* = 0.091 Å^−1^ for the lamellar (*L*_═_) and 0.204 Å^−1^ for the sub‐lamellar (*L*_∥_) structures, respectively, corresponding to the lamellar and sub‐lamellar periodic lengths of 6.90 and 3.08 nm, respectively. These values are consistent with the values (6.04 and 2.85 nm) obtained from the simulation results (Figure 4a). The similar results obtained from both SAXS experiments and simulations can be found in (Figure 4b,c,e,f) for the lamellar‐in‐lamellar structures at higher DDAB concentrations (60 and 70 wt%). However, it is worth noting that some other investigations have uncovered the regular lamellar phases rather than the lamellar‐in‐lamellar phase in DDAB/water binary systems at high concentrations. Zemb et al. observed a lamellar phase with *d* = 3.2 nm (similar to *L*_∥_ phase),^[^ ^30^ ^]^ while Ferreira and Loh identified a lamellar phase with *d* = 6.1 nm (similar to *L*_═_ phase).^[^ ^31^ ^]^ These studies indirectly support the findings of lamellar‐in‐lamellar structures in our work. Furthermore, the hierarchically lamellar structures are confirmed through polarizing optical microscopy (POM) observations. According to Figure 4g–i, the POM images clearly exhibit optically anisotropic birefringence, which displays the typical oily streaks and mosaic textures of a lamellar liquid crystal phase.^[^ ^32^, ^33^, ^34^, ^35^, ^36^ ^]^

Next, the formation mechanism of lamellae‐in‐lamellae in DDAB/water binary mixtures is discussed. The emergence of lamellae‐in‐lamellae in double‐tail cationic surfactant solutions is intriguing and perplexing because similar phenomena are typically observed in block copolymer mixtures or their nanoparticle derivatives on a larger scale^[^ ^32^, ^37^, ^38^, ^39^, ^40^, ^41^, ^42^, ^43^, ^44^ ^]^ but have never been reported in surfactant systems, to our best knowledge. The lamellar phase of the surfactant/water binary systems generally is based on the bilayer as depicted in Figure 3c. Intuitively, in the lamellar‐in‐lamellar structure, the DDAB molecules with large curved angles (173.7°) at the interface should be in direct contact with the aqueous environment, which is energetically unfavorable due to the exposed hydrophobic tails. In fact, to alleviate this issue, the bent DDAB molecules with 145.9° curved angles in the lateral monolayers of the inner lamellae partially shield the hydrophobic tails of the DDAB molecules at the interface, thereby preventing them from direct exposure to water, as shown in Figure 3b. In addition, as suggested by Song and colleagues, the backbone rigidity plays a significant role in maintaining the lamellae‐in‐lamellae structure in ABC‐type bottlebrush copolymer systems.^[^ ^44^ ^]^ In the present DDAB surfactant system, we deem that the molecular chain length is also an important influencing factor. Surfactants with shorter chain lengths have less hydrophobicity, which mitigates the disadvantage of hydrophobic tails being directly exposed to the aqueous environment. Moreover, due to the greater rigidity of short chains, the molecules are less likely to bend, and thus more likely to maintain a large curved angle in the *L*_∥_ lamellar phase. However, surfactants with longer chain lengths, have better flexibility and stronger hydrophobicity, making the overall chains easier to bend and form regular bilayer structures (Figure 3c). To further explore this point, we examined two additional double‐chain surfactant systems, dimethyldioctylammonium bromide (DOAB) with shorter tails (Figure S9a, Supporting Information) and dimethyldipalmitylammonium bromide (DPAB) with longer tails (Figure S10a, Supporting Information), in comparison to DDAB. Figure S9c–e shows that DOAB also forms lamellae‐in‐lamellae and obtains more organized hierarchical structures even at 50 wt% concentration. RDF results (Figure S9b, Supporting Information) demonstrate the existence of this hierarchical structure with a long‐range ordered arrangement, and the spacing (2.10 nm) between two peaks closely matches the length of a fully stretched DOAB molecule. On the contrary, the DPAB system does not exhibit a lamellar‐in‐lamellar phase or any long‐range ordered arrangements (Figure S10, Supporting Information). Additionally, DOAB with a shorter tail length exhibits a larger curved angle, while DPAB with a longer tail length presents a smaller curved angle, as shown in Figure S11 and Table S3 (Supporting Information). This observation solidifies the assertion that molecular rigidity significantly influences the formation of lamellar‐in‐lamellar structures. Additionally, we computed the energy change (Δ*E*) of a transition from a large to a small curved angle in double‐tailed surfactants with varying tail lengths. Our findings reveal that double‐tailed surfactant molecules with short tail lengths (DOAB and DDAB) require more energy to overcome chain tension from large curved angles to small ones, as indicated by their higher Δ*E* values in Table S4 (Supporting Information). In contrast, surfactants with longer tail lengths (DPAB) necessitate comparatively less energy for changing to small curved angles. The stronger intermolecular attractive forces between the longer hydrophobic tails disrupt the balance between the contributions from the molecular rigidity and hydrophilic/hydrophobic interactions. Therefore, surfactants with longer tails, such as DPAB, tend to form regular lamellar structures rather than lamellar‐in‐lamellar structures.

The comparisons described above provide insights into how molecular flexibility alters their self‐assembly behaviors by influencing multi‐scale ordering. In a word, there are two reasonably plausible driving forces behind the formation of this hierarchical layers structure. First, the lateral monolayers of the inner lamellae can prevent the hydrophobic tails of the surfactants at the interface from being directly exposed to the aqueous environment to some extent. Second, surfactants with shorter chain lengths have less hydrophobicity and greater rigidity, which can reduce the direct exposure of the tails and is also conducive to maintaining the chain conformation with a large curved angle. Additionally, compared to the regular lamellar phase (Figure 3c), the lamellar‐in‐lamellar structure can accommodate more surfactant molecules, exhibiting a higher filled density (density of self‐assemblies) in Table 1. This ensures the system maintains a hierarchical arrangement at high surfactant concentrations. Moreover, the higher surfactant concentration is beneficial for forming a more densely packed hydrophobic core to reduce exposure to aqueous surroundings, enhancing the stability of the hierarchically layered structure.

In this work, we also systematically investigate the effects of box sizes, temperatures, and salt concentrations on the self‐assembly behaviors of DDAB solutions. First, in order to demonstrate that our results are independent of the box size, we consider two different simulation boxes with sizes of 24 and 36 nm along the *Z*‐axis direction. As shown in Figure S12 (Supporting Information), the formation of stable lamellar‐in‐lamellar structures in both cases suggests that the finite size effect can be ignored in this work. Second, the simulations are conducted at different temperatures (308 and 318 K) to study the effect of temperature on the formation and stability of self‐assembled structures. The results reveal that the lamellar‐in‐lamellar structures will become unstable or even disappear at elevated temperatures due to the increased thermal energy (Figure S13, Supporting Information). Furthermore, salt concentrations also exert pronounced effects on the self‐assembly behavior. In this work, we consider three different salt concentrations (0.01, 0.05, and 0.1 M NaCl). It is observed that, at higher salt concentrations, the lamellar‐in‐lamellar structures become unstable or even disrupted (Figure S14, Supporting Information). The disruption of the lamellar‐in‐lamellar structure at sufficient high salt concentrations is attributed to the electrostatic screening and hydration effects caused by the mobile ions. The presence of additional ions in the system leads to the Debye screening effect that diminishes the strength of the electrostatic interactions between DDAB molecules. These interactions are crucial for maintaining the hierarchical structures. In addition, the increased ionic strength induced by higher salt concentrations can change the hydration layer surrounding the DDAB molecules (Figure S14h, Supporting Information). This alteration in the hydration shell affects the solvation properties of the molecules, potentially destabilizing the organized packing within the lamellar structure.

## 3 Conclusion

In summary, this work unravels a fascinating hierarchical structure known as “lamellae‐in‐lamellae” in double‐tail cationic surfactant systems. The formation of lamellae‐in‐lamellae is attributed to a multifaceted interplay of factors. A lateral monolayer serves as a protective shield, preventing direct exposure of inner hydrophobic tails to the aqueous environment. Additionally, the less hydrophobicity and greater rigidity of surfactants with shorter chain lengths play a vital role in alleviating the entropy penalty due to the water molecule's rearrangement and maintaining a chain conformation with a large curved angle. Experimental validation through SAXS and POM solidifies these findings. Our work reveals molecular conformations and flexibility can profoundly alter the self‐assembly behavior. Clarifying these fundamental principles of assembly design lays the foundation for engineering multifunctional structured nanomaterials with wide‐range applications.

## 4 Computational Section

### 4.1 System Setup

The mappings of atomistic dimethyldioctylammonium bromide (DOAB, 2C_8_Br), didodecyldimethylammonium bromide (DDAB, 2C_12_Br) and dimethyldipalmitylammonium bromide (DPAB, 2C_16_Br) into coarse‐grained representation are displayed in Figure 1a and Figures S9a and S10a (Supporting Information). The choice of CG particles for these three surfactants is based on previously modeled CG DDAB and the Martini force field.^[^ ^21^, ^22^, ^45^ ^]^ The model for the positively charged surfactants uses Q_0_ beads (representing ionic beads without hydrogen bond donating/accepting abilities) to depict the charged head groups. The alkyl chains are modeled by non‐polar C_2_ beads for the methyl groups connecting the charged head and the alkyl chain, while C_1_ beads are chosen for the rest of the alkyl tails. The negatively charged Br^−^ counterions are represented by Q_a_ beads (ionic hydrogen bond acceptors), implicitly including the effect of six hydrating water molecules around each ion, mimicking the first solvation shell. The standard Martini water model is used, with P_4_ beads representing four implicit water molecules. Additionally, 10% of antifreeze “big” BP_4_ water beads are added, disrupting close packing of the uniform bead sizes, since the σ value of the Lennard–Jones potential for ${\mathrm{BP}}_{4}-P_{4}$ interactions is 0.57 nm rather than the 0.47 nm for P_4_ − P_4_, preventing artificial freezing of the water phase. Detailed definitions of the nature of CG particle types are illustrated by Marrink et al.^[^ ^22^ ^]^ PACKMOL software is used to construct the initial random systems.^[^ ^46^ ^]^ A summary of the 2C_*n*_Br/water systems is illustrated in Tables S1 and S2 (Supporting Information).

### 4.2 Details of the Molecular Dynamics Simulation

All the simulations are performed in the isothermal‐isobaric (NPT) ensemble using GROMACS 2021.4 under periodic boundary conditions in three directions.^[^ ^47^, ^48^ ^]^ The van der Waals interaction is described by the Lennard–Jones potential with a truncation radius of 1.1 nm. The electrostatic interaction is treated by the Particle Mesh Ewald (PME) method with a cutoff of 1.1 nm.^[^ ^49^ ^]^ To begin with, the steepest descent algorithm is employed to eliminate the unreasonable structure of the initial system before simulation.^[^ ^50^ ^]^ The target pressure is set to 1.01325 bar for all NPT simulations. Then a 100 ns annealing process (298 K → 350 K → 298 K) is undertaken on each system with a Berendsen scheme to control temperature and pressure.^[^ ^51^ ^]^ Subsequently, a 3.75 µ*s* simulation is performed to ensure the equilibrium of the system, using the Bussi–Donadio–Parrinello thermostat and the Berendsen barostat.^[^ ^51^, ^52^ ^]^ Finally, a 250 ns simulation is performed and sampled, and the systems are maintained at 298 K and 1.01325 bar controlled by Bussi–Donadio–Parrinello thermostat and Parrinello–Rahman semiisotropic barostat, respectively.^[^ ^53^, ^54^ ^]^ A time step of 25.0 fs is applied in all simulations except for the annealing process with a 20.0 fs time step. For considering the impact of other temperatures, the value of 298 K changed to the desired one. The OVITO and VMD software are performed for visualization of MD trajectories.^[^ ^55^, ^56^ ^]^

### 4.3 Data Analyses

#### 4.3.1 Critical Micelle Concentration

Critical micelle concentration (CMC) is determined by calculating the average concentration of free surfactants after the system reaches equilibrium.^[^ ^57^, ^58^ ^]^ This process involves identifying surfactants as “free” when they are not part of an aggregate, a decision based on the aggregate size distribution, typically by choosing a cutoff size that lies between two prominent peaks in the distribution. A 100 ns annealing process (298 K → 350 K → 298 K) is undertaken on each system with a Berendsen scheme to control temperature and pressure.^[^ ^51^ ^]^ Subsequently, a 250 ns simulation is performed to ensure the equilibrium of the system, using the Bussi–Donadio–Parrinello thermostat and the Berendsen barostat.^[^ ^51^, ^52^ ^]^ Finally, a 250 ns simulation is performed and sampled, and the systems are maintained at 298 K and 1.01325 bar controlled by Bussi–Donadio–Parrinello thermostat and Parrinello–Rahman semiisotropic barostat, respectively.^[^ ^53^, ^54^ ^]^ A time step of 25.0 fs is applied in all simulations except for the annealing process with a 20.0 fs time step. During these simulations, the quantity of free surfactants is monitored and counted at each time point, with their equilibrium average concentration representing the CMC. For a more effective comparison, the experimentally obtained CMC values for DOAB (19.8 mM), DDAB (0.5 mM), and DPAB (0.007 mM), as cited in the literature,^[^ ^23^, ^24^ ^]^ are represented as a distinct line in Figure S1a–c (Supporting Information).

#### 4.3.2 Radial Distribution Function

Radial Distribution Function (RDF) or pair correlation function *g*(*r*) between particles of type *A* and *B* is defined in the following way:

(1) $$g_{AB}\left(r\right)=\frac{\langle \rho_{B}\left(r\right)\rangle }{{\langle \rho_{B}\rangle }_{\text{local}\;}}=\frac{1}{{\langle \rho_{B}\rangle }_{\text{local}\;}}\frac{1}{N_{A}}\sum_{i\in A}^{N_{A}}\sum_{j\in B}^{N_{B}}\frac{\delta \left(r_{ij}-r\right)}{4\pi r^{2}}$$

with 〈ρ_*B*_(*r*)〉 the particle density of type *B* at a distance *r* around particles *A*, and 〈ρ_*B*_〉_local_ the particle density of type *B* averaged over all spheres around particles *A* with radius *r*_max _.

#### 4.3.3 Structure Factor

Structure factor (S(q)) calculations are performed by

(2) $$S\left(q\right)=\frac{{\left(\sum_{j}\cos\left(q\cdot r_{j}\right)\right)}^{2}+{\left(\sum_{j}\sin\left(q\cdot r_{j}\right)\right)}^{2}}{N}$$

where q is the scattering vector, the $r_{j}$ are the position vectors, and *N* is the number of beads. The scattering vector, q, is restricted to integer numbers of wavelengths within the simulation box, that is, $q=2\pi (n_{x}/L_{x}$, *n*_*y*_/*L*_*y*_, *n*_*z*_/*L*_*z*_).^[^ ^59^, ^60^, ^61^ ^]^

#### 4.3.4 Filled Density

Filled density (ρ_f_) is undertaken by

(3) $$\rho_{f}=\frac{M_{\text{sur}}}{V_{\text{sur}}}$$

The total mass of the surfactant is given by M_sur_, and V_sur_ refers to the occupied surfactant volume (filled volume) in the system. This filled volume is computed by constructing the surfactant/water interface from millions of triangular mesh faces using the alpha shape method in OVITO.^[^ ^62^ ^]^ The volume enclosed by this surface corresponds to the total filled volume.

#### 4.3.5 Single Point Energy

Single point energy of surfactant molecule with different curved angles is calculated in Gaussian 16 package.^[^ ^63^ ^]^ This commenced with an initial geometry optimization and frequency analysis, utilizing the B3LYP/6‐311G(d) theoretical framework coupled with SMD solvent modeling. Subsequent to this, we conducted single‐point energy calculations applying the M062X/6‐311G(d,p) method. This rigorous approach enabled a thorough examination of the surfactant molecule's structural, energetic, and vibrational attributes, particularly focusing on how these properties evolve with changes in molecular curvature, all within a simulated aqueous environment.

## 5 Experimental Section

### Materials and Sample Preparation

DDAB (purity = 99%, M = 462.63 g mol^−1^) was purchased from Acros Organics and used without further purification. Ultrapure water with a resistivity above 18.2 MΩ cm^−1^ was obtained by a Milli‐Q system (Millipore®) equipped with a sterile 0.22 µm membrane filter and was used as a solvent. DDAB/water binary mixtures with different weight fractions (50, 60, and 70 wt%) were prepared by weighting appropriate amounts of DDAB powders and mixing them with water by vortex for 10 min at room temperature (24 °C) and waiting for 1 h to allow hydrations of the DDAB crystals to form liquid crystallites. The mixtures were then heated to 40 °C for 5 h for further dissolution and cooled down to room temperature. Afterward, centrifugation at 10000 rpm was made, back and forth, to remove bubbles and to further homogenize the samples, which were equilibrated for at least 30 days at room temperature before characterization.

### Small Angle X‐ray Scattering

Small‐angle X‐ray scattering (SAXS) experiments were performed on a Xenocs Xeuss 2.0 instrument, equipped with JJ X‐ray system Aps pinhole, an X‐ray microsource with a wavelength λ = 1.5406 Å, and a movable 2D 300k PILATUS3 R detector (DECTRIS Ltd, Switzerland). Data were collected at a q range of 0.27 to 14.2 nm^−1^, where the scattering vector *q* = (4π/λ)*sin*(θ/2), and θ is the scattering angle between the scattered beam and the incoming beam. Samples were put in disposable borosilicate glass capillaries and equilibrated for at least 12 h before the experiments which were performed at room temperature (24 °C). Measured scattering intensities (I(q)) of the DDAB/water liquid crystal were put to absolute scales using a built‐in calibration procedure of the instrument and were not subtracted by the contribution of the solvent (water). The measurements were repeated at different times after sample preparation (7 days and 2.5 months) to ensure the equilibrium state of the investigated systems.

### Polarized Optical Microscopy

The textures of liquid crystalline phases of DDAB/water mixtures were observed by polarized optical microscopy (POM, Olympus BX51) at room temperature (24 °C). The textures of investigated systems were compared with the typical textures of surfactant lamellar phases.

## Conflict of Interest

The authors declare no conflict of interest.

## Supporting information

Supporting Information

## Data Availability

The data that support the findings of this study are available from the corresponding author upon reasonable request.

## References

[1] D. Nepal , S. Kang , K. M. Adstedt , K. Kanhaiya , M. R. Bockstaller , L. C. Brinson , M. J. Buehler , P. V. Coveney , K. Dayal , J. A. El‐Awady , L. C. Henderson , D. L. Kaplan , S. Keten , N. A. Kotov , G. C. Schatz , S. Vignolini , F. Vollrath , Y. Wang , B. I. Yakobson , V. V. Tsukruk , H. Heinz , Nat. Mater. 2023, 22, 18. 36446962 10.1038/s41563-022-01384-1

[2] C. L. Hedegaard , A. Mata , Biofabrication 2020, 12, 032002. 32476660 10.1088/1758-5090/ab84cb

[3] Z. Sun , T. Liao , K. Liu , L. Jiang , J. H. Kim , S. X. Dou , Small 2014, 10, 3001. 24753310 10.1002/smll.201400516

[4] J. Zhang , Y. Wang , B. J. Rodriguez , R. Yang , B. Yu , D. Mei , J. Li , K. Tao , E. Gazit , Chem. Soc. Rev. 2022, 51, 6936. 35861374 10.1039/d2cs00122e

[5] E. A. Margolis , N. E. Friend , M. W. Rolle , E. Alsberg , A. J. Putnam , Trends Biotechnol. 2023, 41, 1400. 37169690 10.1016/j.tibtech.2023.04.003 PMC10593098

[6] Y. Liu , D. Luo , T. Wang , Small 2016, 12, 4611. 27322951 10.1002/smll.201600626

[7] C. F. J. Faul , Acc. Chem. Res. 2014, 47, 3428. 25191750 10.1021/ar500162a

[8] K. Miszta , J. de Graaf , G. Bertoni , D. Dorfs , R. Brescia , S. Marras , L. Ceseracciu , R. Cingolani , R. van Roij , M. Dijkstra , L. Manna , Nat. Mater. 2011, 10, 872. 21946613 10.1038/nmat3121

[9] C. Yuan , W. Ji , R. Xing , J. Li , E. Gazit , X. Yan , Nat. Rev. Chem. 2019, 3, 567. 10.1038/s41570-019-0129-8 PMC7617026 39649433

[10] H. Qiu , Z. M. Hudson , M. A. Winnik , I. Manners , Science 2015, 347, 1329. 25792323 10.1126/science.1261816

[11] O. S. Andersen , R. E. Koeppe , Annu. Rev. Biophys. Biomol. Struct. 2007, 36, 107. 17263662 10.1146/annurev.biophys.36.040306.132643

[12] T. Zeng , R. P. Gautam , D. H. Ko , H.‐L. Wu , A. Hosseini , Y. Li , C. J. Barile , E. C. M. Tse , Nat. Rev. Chem. 2022, 6, 862. 37117701 10.1038/s41570-022-00433-2

[13] N. Tran , J. Zhai , C. E. Conn , X. Mulet , L. J. Waddington , C. J. Drummond , J. Phys. Chem. Lett. 2018, 9, 3397. 29809009 10.1021/acs.jpclett.8b01110

[14] K. J. Clinckspoor , F. B. Okasaki , E. Sabadini , J. Colloid Interface Sci. 2022, 607, 1014. 34571291 10.1016/j.jcis.2021.09.018

[15] T. Wang , S. Dai , J. Wang , B. Liu , M. Cao , B. Guan , Y. Han , Y. Wang , Nano Res. 2023, 16, 2551.

[16] D. Benkowska‐Biernacka , S. G. Mucha , L. Firlej , F. Formalik , J.‐L. Bantignies , E. Anglaret , M. Samoć , K. Matczyszyn , ACS Appl. Mater. Interfaces 2023, 15, 32717. 37366586 10.1021/acsami.3c05656 PMC10347123

[17] L. Peng , H. Peng , Y. Liu , X. Wang , C.‐T. Hung , Z. Zhao , G. Chen , W. Li , L. Mai , D. Zhao , Sci. Adv. 2021, 7, 7403. 10.1126/sciadv.abi7403 PMC8565844 34730995

[18] H. K. Murnen , A. M. Rosales , J. N. Jaworski , R. A. Segalman , R. N. Zuckermann , J. Am. Chem. Soc. 2010, 132, 16112. 20964429 10.1021/ja106340f

[19] Q. Liu , Z. Sun , Y. Dou , J. H. Kim , S. X. Dou , J. Mater. Chem. A 2015, 3, 11688.

[20] Y. Cao , S. Yang , Y. Li , J. Shi , Aggregate 2021, 2, 49.

[21] D. Patel , G. Pérez‐Sánchez , M. Jorge , D. Ray , V. K. Aswal , K. Kuperkar , J. A. P. Coutinho , P. Bahadur , Langmuir 2023, 39, 2692. 36763753 10.1021/acs.langmuir.2c03176

[22] S. J. Marrink , H. J. Risselada , S. Yefimov , D. P. Tieleman , A. H. de Vries , J. Phys. Chem. B 2007, 111, 7812. 17569554 10.1021/jp071097f

[23] H. Li , Z. Cai , Y. Wang , Langmuir 2020, 36, 14113. 33166156 10.1021/acs.langmuir.0c02783

[24] H. Li , Z. Liu , C. Li , Q. Feng , Y. Liu , Q. Li , Z. Dong , Y. Wang , L. Jiang , J. Mater. Chem. A 2020, 8, 17392.

[25] Y. Xu , W. Li , F. Qiu , Y. Yang , A.‐C. Shi , J. Phys. Chem. B 2010, 114, 14875. 21028862 10.1021/jp1068335

[26] V. Markov , Y. Kriksin , I. Erukhimovich , G. ten Brinke , J. Chem. Phys. 2013, 139, 084906. 24007036 10.1063/1.4818872

[27] Z. Zhong , G. Du , Y. Wang , J. Jiang , Langmuir 2023, 39, 11081. 37493456 10.1021/acs.langmuir.3c01410

[28] A. G. Moreira , R. R. Netz , Phys. Rev. Lett. 2001, 87, 078301. 11497925 10.1103/PhysRevLett.87.078301

[29] L. Herrmann , A. Johner , P. Kékicheff , Phys. Rev. Lett. 2014, 113, 268302. 25615394 10.1103/PhysRevLett.113.268302

[30] T. Zemb , D. Gazeau , M. Dubois , T. Gulik‐Krzywicki , Europhys. Lett. 1993, 21, 759.

[31] G. A. Ferreira , W. Loh , J. Braz. Chem. Soc. 2016, 27, 392.

[32] A. H. Hofman , M. Reza , J. Ruokolainen , G. ten Brinke , K. Loos , Macromolecules 2014, 47, 5913.

[33] S. Hanski , N. Houbenov , J. Ruokolainen , D. Chondronicola , H. Iatrou , N. Hadjichristidis , O. Ikkala , Biomacromolecules 2006, 7, 3379. 17154466 10.1021/bm0606770

[34] S. Koitani , S. Dieterich , N. Preisig , K. Aramaki , C. Stubenrauch , Langmuir 2017, 33, 12171. 29028344 10.1021/acs.langmuir.7b02101

[35] Y. Zhao , X. Yue , X. Wang , X. Chen , J. Colloid Interface Sci. 2013, 389, 199. 23062960 10.1016/j.jcis.2012.09.032

[36] F. Caboi , M. Monduzzi , Langmuir 1996, 12, 3548.

[37] R. Liang , Y. Xue , X. Fu , A. N. Le , Q. Song , Y. Qiang , Q. Xie , R. Dong , Z. Sun , C. O. Osuji , J. A. Johnson , W. Li , M. Zhong , Nat. Mater. 2022, 21, 1434. 36357688 10.1038/s41563-022-01393-0

[38] R. Nap , N. Sushko , I. Erukhimovich , G. ten Brinke , Macromolecules 2006, 39, 6765.

[39] A. Subbotin , T. Klymko , G. ten Brinke , Macromolecules 2007, 40, 2915.

[40] A. H. Hofman , G. ten Brinke , K. Loos , Polymer 2016, 107, 343.

[41] A. H. Hofman , M. Reza , J. Ruokolainen , G. ten Brinke , K. Loos , Angew. Chemie Int. Ed. 2016, 55, 13081. 10.1002/anie.201606890 PMC5113798 27633842

[42] X. Zhang , L. Wang , L. Zhang , J. Lin , T. Jiang , Langmuir 2015, 31, 2533. 25654644 10.1021/la503985u

[43] G. Fleury , F. S. Bates , Macromolecules 2009, 42, 1691.

[44] Q. Song , Q. Dong , R. Liang , Y. Xue , M. Zhong , W. Li , Macromolecules 2023, 56, 5470.

[45] T. D. Potter , E. L. Barrett , M. A. Miller , J. Chem. Theory Comput. 2021, 17, 5777. 34472843 10.1021/acs.jctc.1c00322 PMC8444346

[46] L. Martinez , R. Andrade , E. G. Birgin , J. M. Martínez , J. Comput. Chem. 2009, 30, 2157. 19229944 10.1002/jcc.21224

[47] E. Lindahl , M. Abraham , B. Hess , D. van der Spoel , Gromacs 2021.4 source code, 2021, 10.5281/zenodo.5636567.

[48] M. J. Abraham , T. Murtola , R. Schulz , S. Páll , J. C. Smith , B. Hess , E. Lindah , SoftwareX 2015, 1‐2, 19.

[49] U. Essmann , L. Perera , M. L. Berkowitz , T. Darden , H. Lee , L. G. Pedersen , J. Chem. Phys. 1995, 103, 8577.

[50] Y. Wardi , J. Optim. Theory Appl. 1988, 59, 307.

[51] H. J. C. Berendsen , J. P. M. Postma , W. F. van Gunsteren , A. DiNola , J. R. Haak , J. Chem. Phys. 1984, 81, 3684.

[52] G. Bussi , D. Donadio , M. Parrinello , J. Chem. Phys. 2007, 126, 014101. 17212484 10.1063/1.2408420

[53] M. Parrinello , A. Rahman , J. Appl. Phys. 1981, 52, 7182.

[54] S. Nosé , M. Klein , Mol. Phys. 1983, 50, 1055.

[55] A. Stukowski , Model. Simul. Mater. Sci. Eng. 2010, 18, 015012.

[56] W. Humphrey , A. Dalke , K. Schulten , J. Mol. Graph. 1996, 14, 33. 8744570 10.1016/0263-7855(96)00018-5

[57] B. G. Levine , D. N. LeBard , R. DeVane , W. Shinoda , A. Kohlmeyer , M. L. Klein , J. Chem. Theory Comput. 2011, 7, 4135. 26598358 10.1021/ct2005193

[58] S. A. Sanders , A. Z. Panagiotopoulos , J. Chem. Phys. 2010, 132, 11. 10.1063/1.3358354 20331315

[59] Y. Sun , P. Padmanabhan , M. Misra , F. A. Escobedo , Soft Matter 2017, 13, 8542. 29095474 10.1039/c7sm01819c

[60] A. A. Gavrilov , Y. V. Kudryavtsev , P. G. Khalatur , A. V. Chertovich , Chem. Phys. Lett. 2011, 503, 277.

[61] T. Mabuchi , S.‐F. Huang , T. Tokumasu , Macromolecules 2020, 53, 3273.

[62] A. Stukowski , JOM 2014, 66, 399.

[63] M. J. Frisch , G. W. Trucks , H. B. Schlegel , G. E. Scuseria , M. A. Robb , J. R. Cheeseman , G. Scalmani , V. Barone , G. A. Petersson , H. Nakatsuji , X. Li , M. Caricato , A. V. Marenich , J. Bloino , B. G. Janesko , R. Gomperts , B. Mennucci , H. P. Hratchian , J. V. Ortiz , A. F. Izmaylov , J. L. Sonnenberg , D. Williams‐Young , F. Ding , F. Lipparini , F. Egidi , J. Goings , B. Peng , A. Petrone , T. Henderson , D. Ranasinghe , et al., Gaussian ∼16 Revision C.01, Gaussian Inc., Wallingford CT 2016.

