# Supplementary material for: Self‐Assembly of Lamellae‐in‐Lamellae by Double‐Tail Cationic Surfactants
Source: Adv Sci (Weinh). 2024 May 15;11(28):2401210. doi: 10.1002/advs.202401210 (PMC11267300; doi:10.1002/advs.202401210)
Supplement: Supplementary file 1 — Supporting Information [file ADVS-11-2401210-s001.pdf]

ADVANCED SCIENCE
Open Access

Supporting Information

for *Adv. Sci.*, DOI 10.1002/advs.202401210

Self-Assembly of Lamellae-in-Lamellae by Double-Tail Cationic Surfactants

Zhixuan Zhong, Guanqun Du, Linbo Ma, Yilin Wang and Jian Jiang**

Supporting Information

for

Self-Assembly of Lamellae-in-Lamellae by Double-Tail Cationic Surfactants

Zhixuan Zhong#,[†,‡] Guanqun Du#,[¶] Linbo Ma,[†,‡]

Yilin Wang,[*,¶,‡] and Jian Jiang[*,†,‡]

†*Beijing National Laboratory for Molecular Sciences, State Key Laboratory of Polymer Physics and Chemistry, Institute of Chemistry, Chinese Academy of Sciences, Beijing 100190, P. R. China*

‡*University of Chinese Academy of Sciences, Beijing 100049, P. R. China*

¶*CAS Key Laboratory of Colloid, Interface and Chemical Thermodynamics, CAS Research/Education Center for Excellence in Molecular Sciences, Beijing National Laboratory for Molecular Science, Institute of Chemistry, Chinese Academy of Sciences, Beijing 100190, P. R. China*

E-mail: yilinwang@iccas.ac.cn; jiangj@iccas.ac.cn

# These authors contributed equally to this work.

Supporting Figures and Tables

Table S1: Summary of DDAB system.

wt%	$N_{surfactant}$	N_{water}	N_{NaCl}	Box Size (nm^3)	Temperature (K)
1	270	213840	-	30 * 30 * 30	298
5	1350	205200	-	30 * 30 * 30	298
20	5400	172800	-	30 * 30 * 30	298
50	13500	108000	-	30 * 30 * 30	298
60	16200	86400	-	30 * 30 * 30	298
70	18900	64800	-	30 * 30 * 30	298
60	12960	69120	-	30 * 30 * 24	298
60	19440	103680	-	30 * 30 * 36	298
60	16200	86400	-	30 * 30 * 30	308
60	16200	86400	-	30 * 30 * 30	318
60	16200	86400	163 (0.01 M)	30 * 30 * 30	298
60	16200	86400	813 (0.05 M)	30 * 30 * 30	298
60	16200	86400	1625 (0.1 M)	30 * 30 * 30	298

Table S2: Summary of DOAB and DPAB/water binary system.

Surfactant	wt%	$N_{surfactant}$	N_{water}	Box Size (nm^3)	Temperature (K)
DOAB	50	18000	108000	30 * 30 * 30	298
	60	21600	86400	30 * 30 * 30	298
	70	25200	64800	30 * 30 * 30	298
DPAB	50	10800	108000	30 * 30 * 30	298
	60	12960	86400	30 * 30 * 30	298
	70	15120	64800	30 * 30 * 30	298

Table S3: Average curved angle of double tail cationic surfactant/water binary systems.

wt%	DOAB	DDAB	DPAB
50	$148.04° \pm 0.18°$	$125.66° \pm 0.36°$	$75.31° \pm 0.28°$
60	$157.12° \pm 0.21°$	$146.88° \pm 0.19°$	$87.47° \pm 0.32°$
70	$154.91° \pm 0.31°$	$152.48° \pm 0.18°$	$98.68° \pm 0.34°$

Table S4: Single point energy of double-tail surfactant molecule with different curved angle.

Surfactant	$E_{152.48°}$ (kJ/mol)	$E_{98.68°}$ (kJ/mol)	ΔE (kJ/mol)
DOAB	-2008218.22	-2008188.48	29.74
DDAB	-2834227.14	-2834203.64	23.50
DPAB	-3660235.35	-3660218.04	17.31

$$\Delta E = E_{98.68°} - E_{152.48°}$$

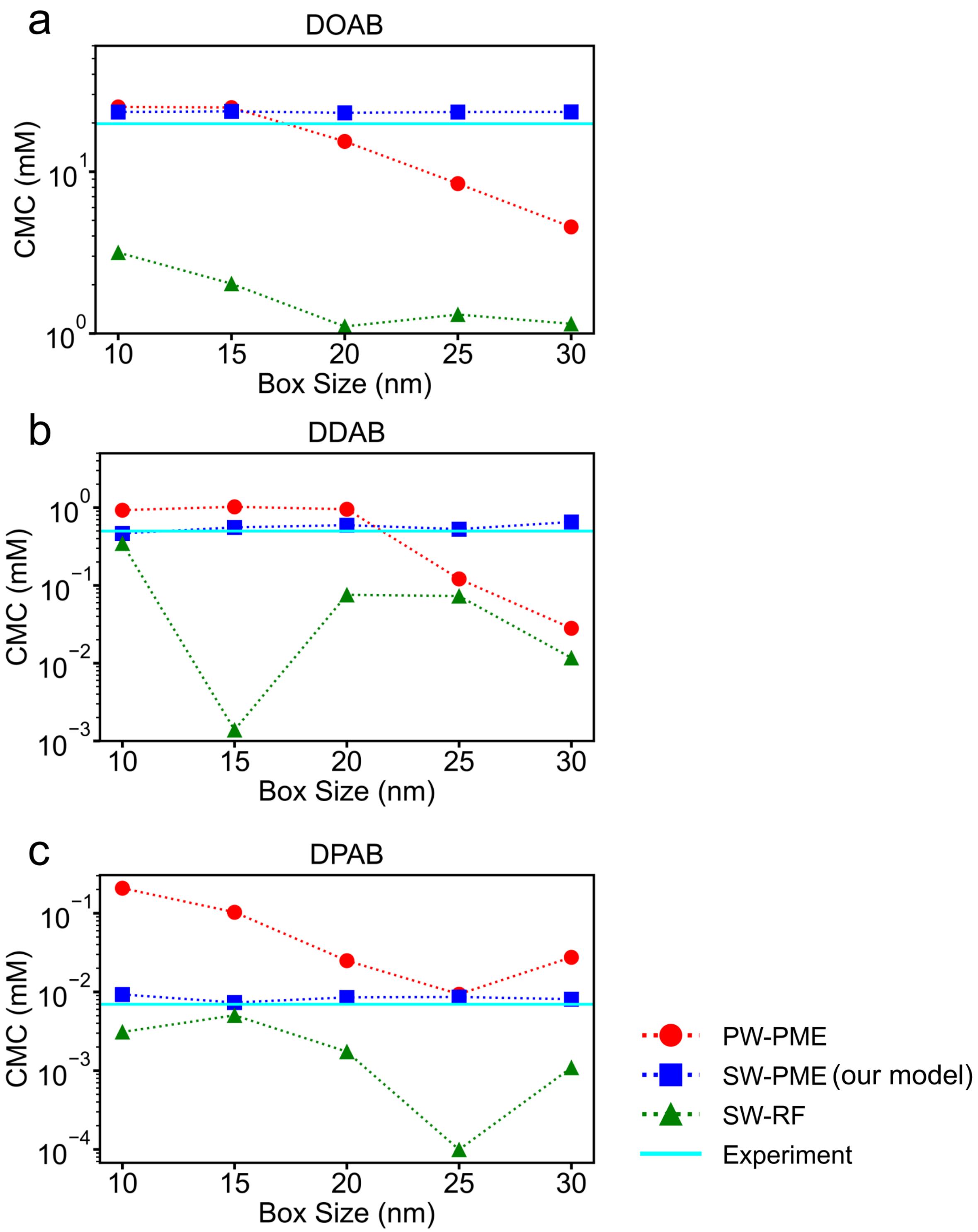

Figure S1. Critical micelle concentration of double-tail surfactant molecule with different tail length. SW represents the standard water model,[1] and the PW refers to the polarizable water model.[2] PME is the Particle Mesh Ewald algorithm and RF is the Reaction Field algorithm. Both algorithms are using for computing the long-range electrostatic interaction.

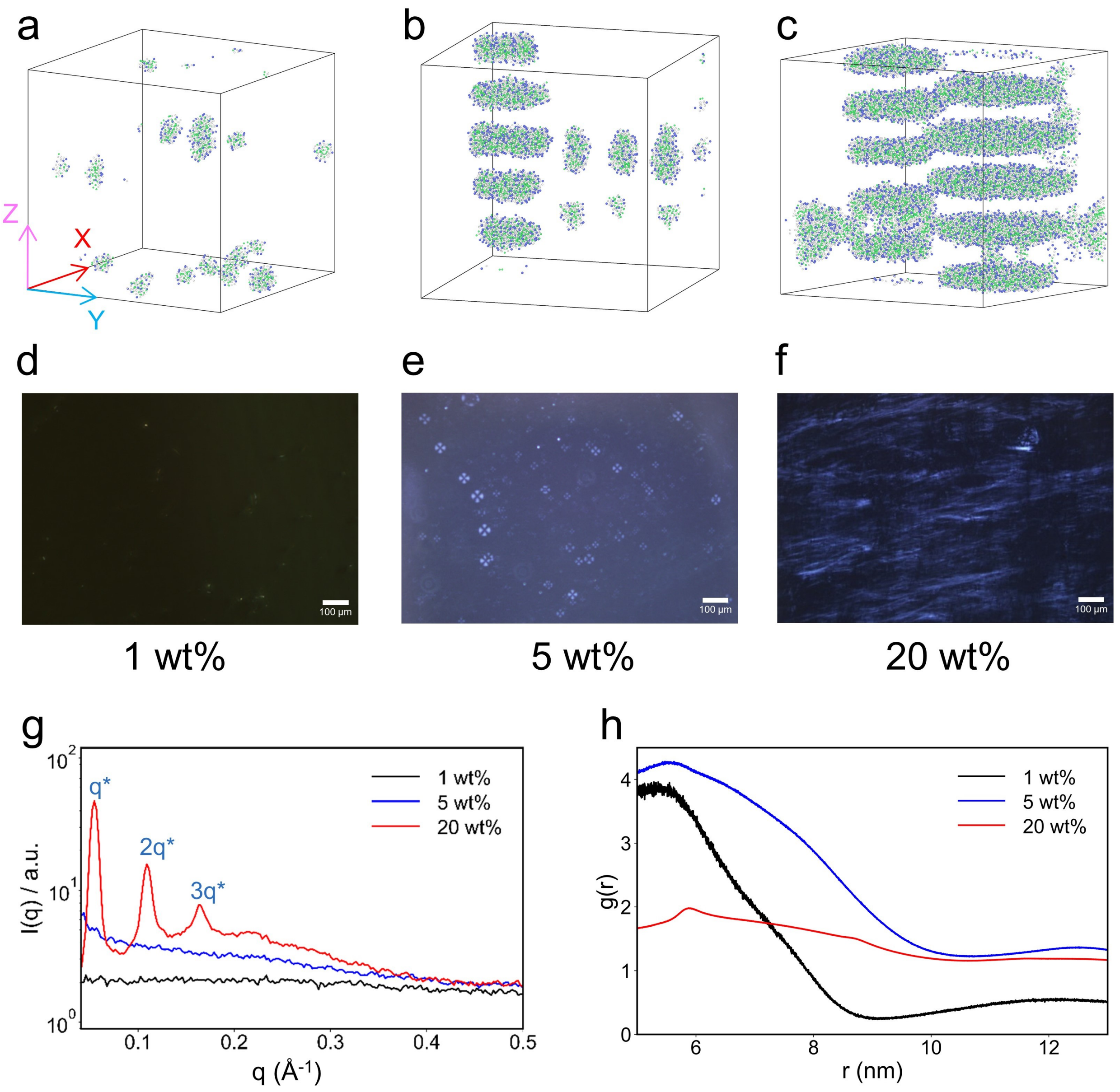

Figure S2. (a-c) Snapshots and (d-f) POM images of (a, d) 1 wt%, (b, e) 5 wt%, and (c, f) 20 wt% DDAB/water binary systems; (g) SAXS profiles and (h) RDF profiles between headgroups of the corresponding dilute DDAB/water binary systems. As the figures shows, the lamellar-in-lamellar structure is not observed in these lower concentrations. This outcome suggests that the formation and stability of the lamellar-in-lamellar structure are highly dependent on the DDAB concentration.

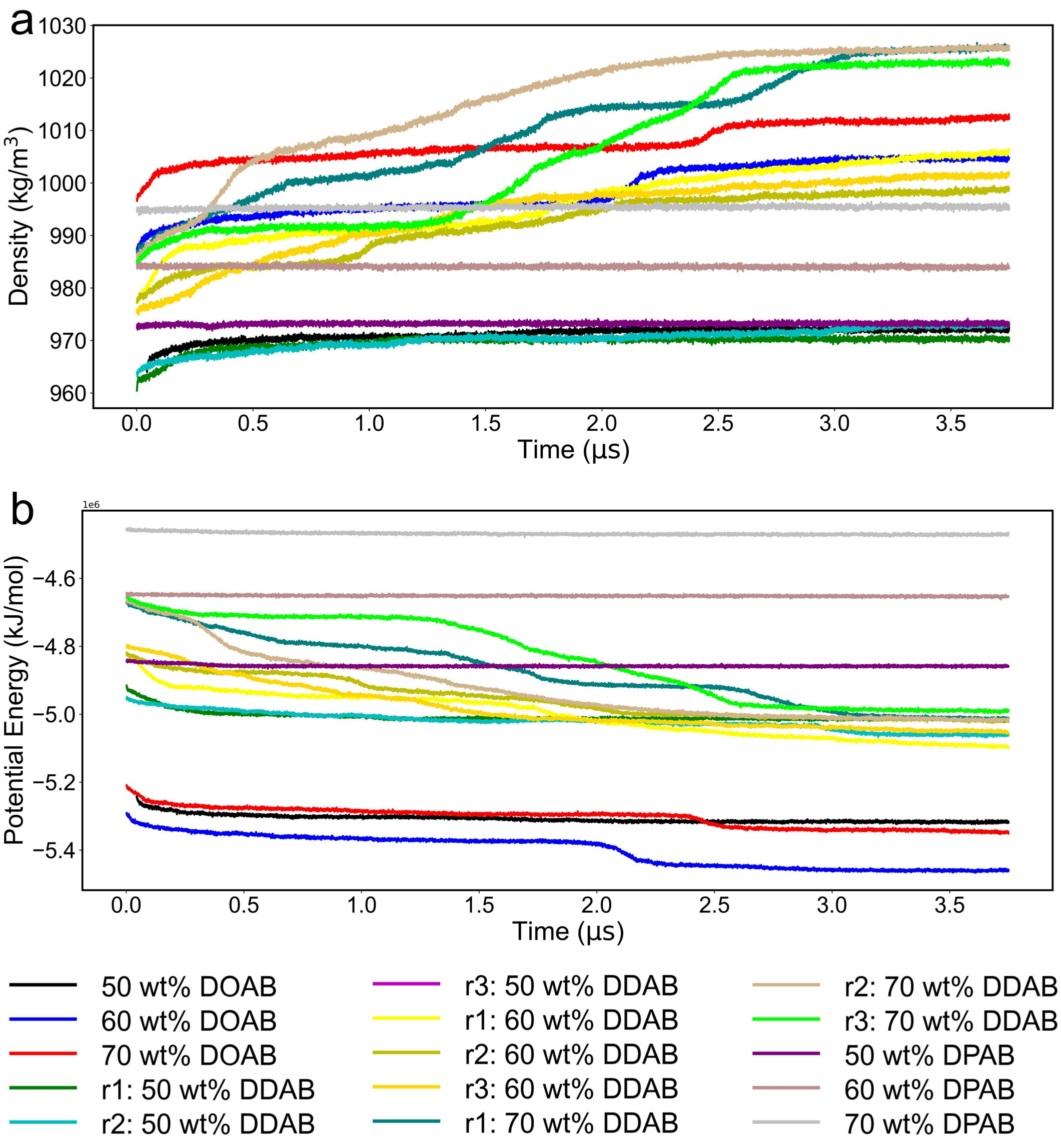

Figure S3. (a) Density and (b) potential energy of different systems as a function of the simulation time. The r1, r2 and r3 represent the replica-1, replica-2 and replica-3, respectively.

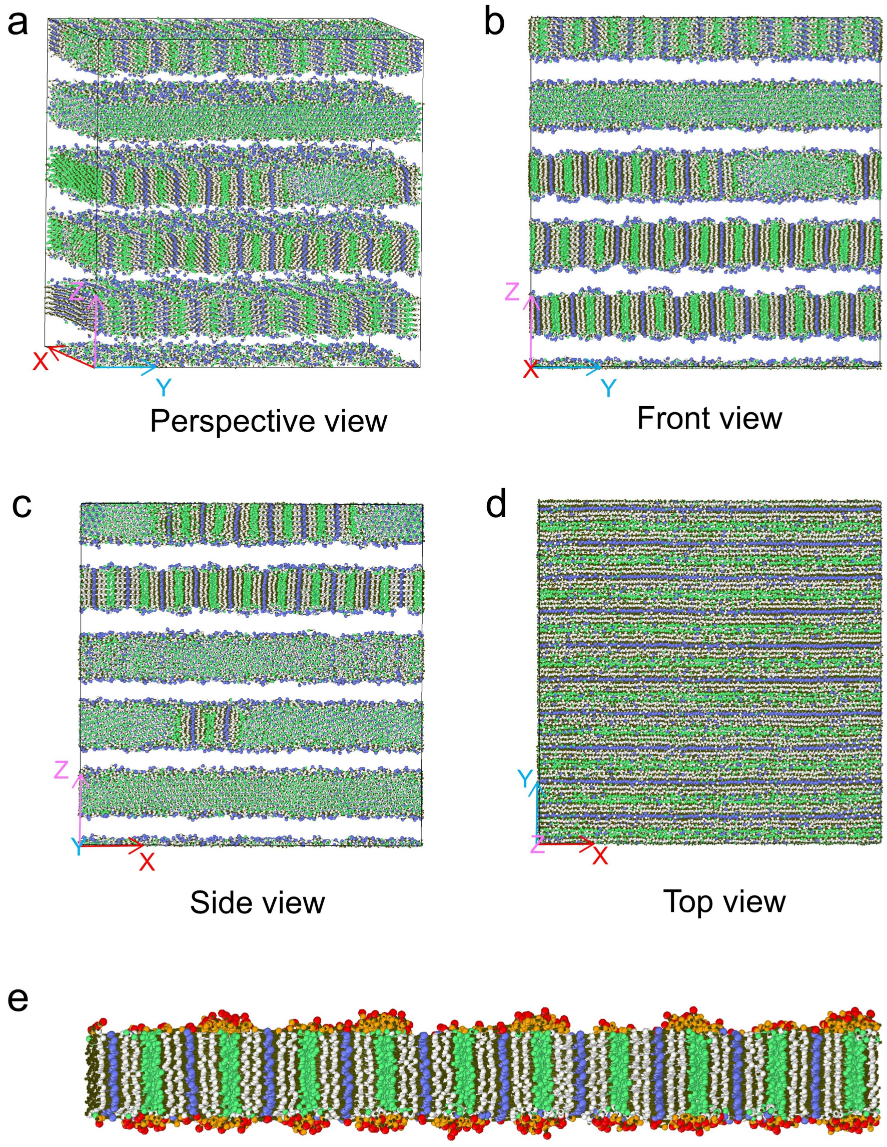

Figure S4. Snapshots of 70 wt% DDAB/water binary system from (a) perspective view, (b) front view, (c) side view, and (d) top view; (e) snapshot of singular layer of 70 wt% DDAB system, where the red beads denote the headgroups (Q_0) of the surfactant's outer monolayer, while the orange beads represent the tails (C_1 and C_2) of the surfactant's outer monolayer.

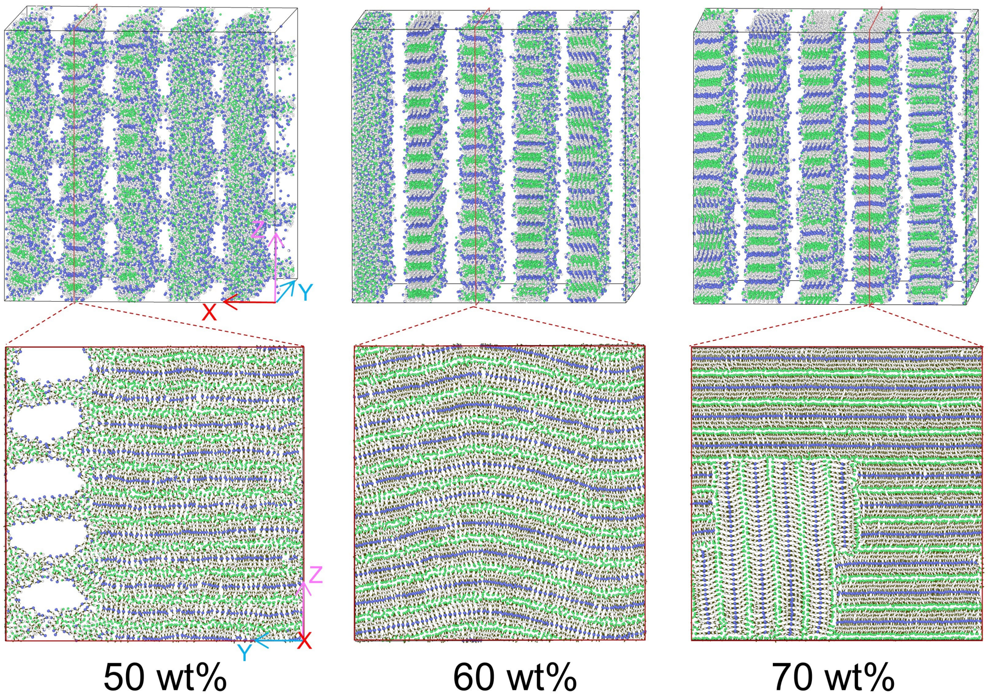

Figure S5. Replica-2: snapshots of 50 wt%, 60 wt% and 70 wt% DDAB/water binary systems.

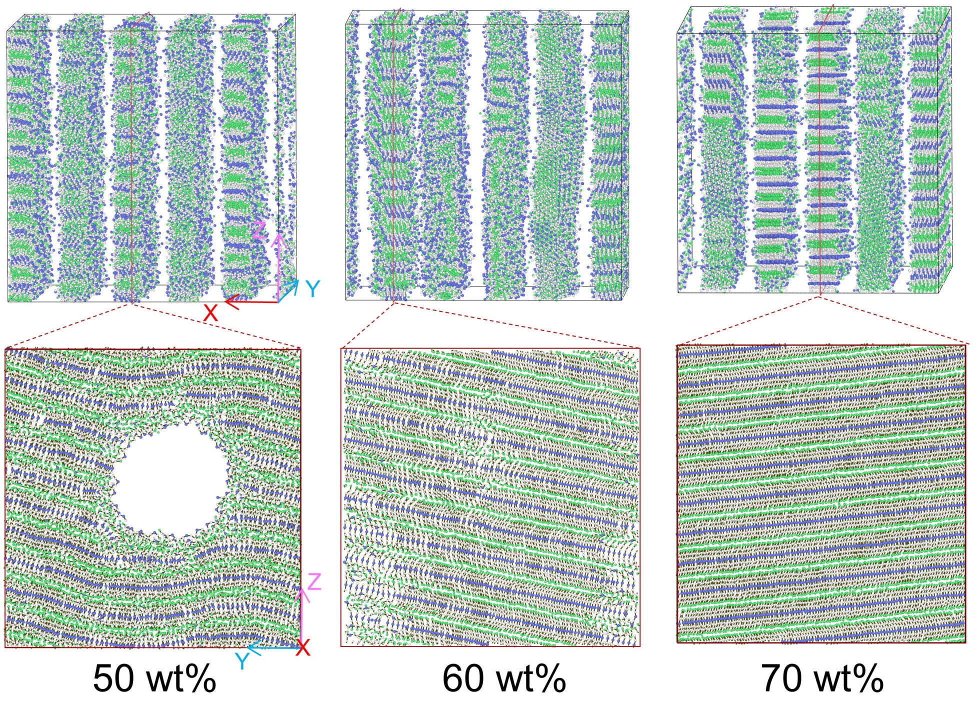

Figure S6. Replica-3: snapshots of 50 wt%, 60 wt% and 70 wt% DDAB/water binary systems.

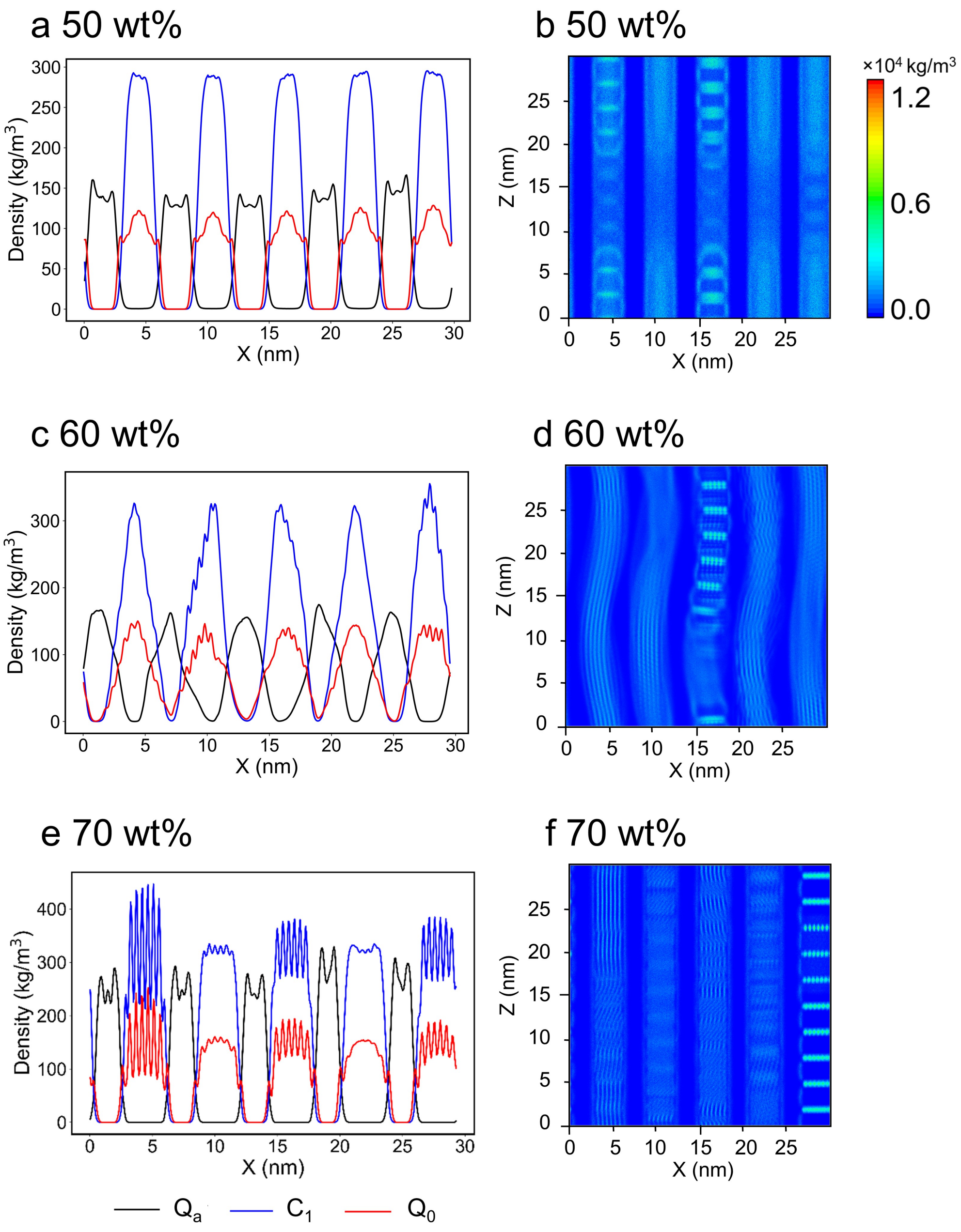

Figure S7. Replica-2: 1D density distribution of (a) 50 wt%, (c) 60 wt% and (e) 70 wt% DDAB/water binary systems; Q_a is the bromine ion bead, C_1 is the terminal bead of each tail of DDAB, and Q_0 is the headgruop bead of DDAB; 2D density maps of headgroup of (b) 50 wt%, (d) 60 wt% and (f) 70 wt% DDAB/water binary systems.

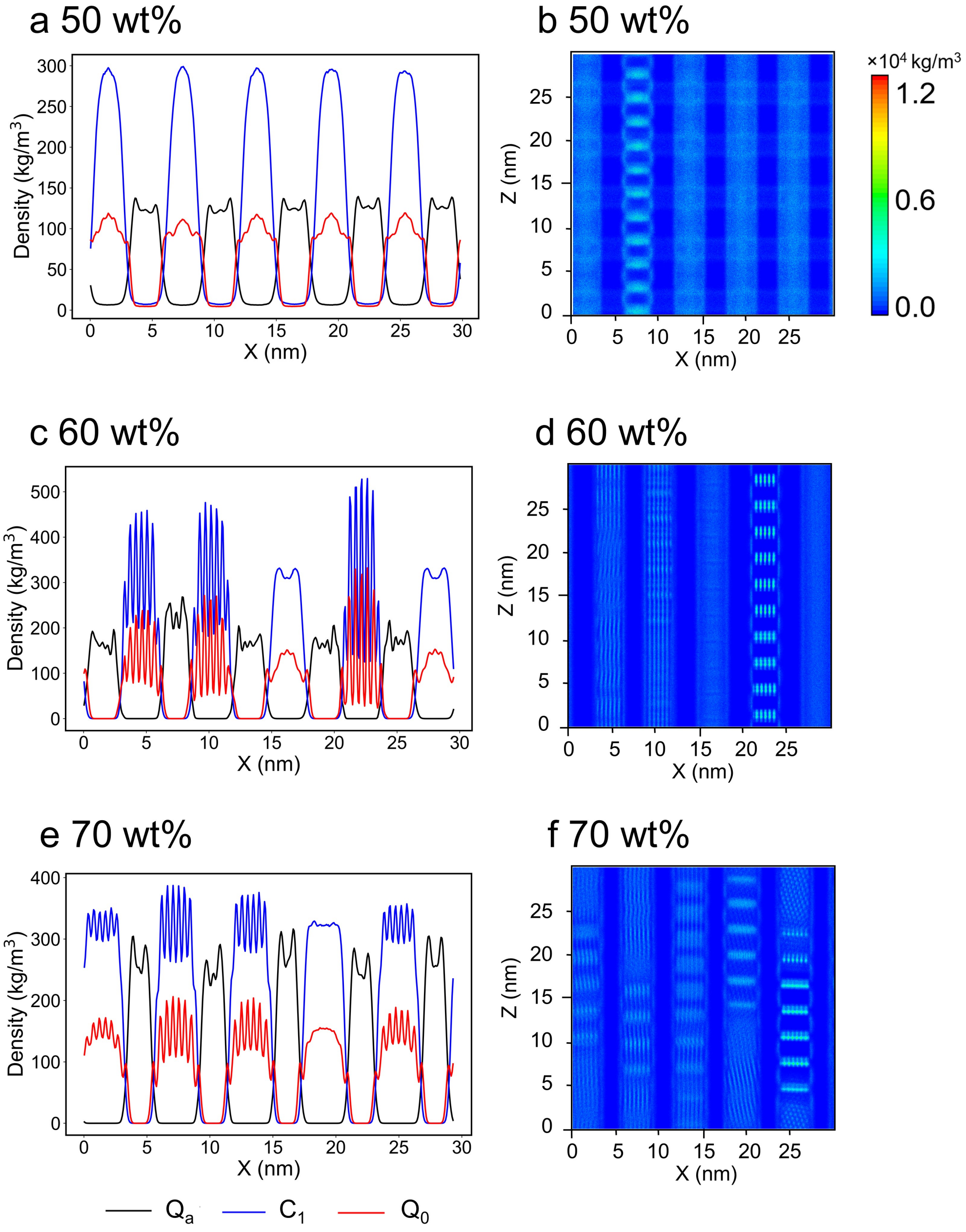

Figure S8. Replica-3: 1D density distribution of (a) 50 wt%, (c) 60 wt% and (e) 70 wt% DDAB/water binary systems; Q_a is the bromine ion bead, C_1 is the terminal bead of each tail of DDAB, and Q_0 is the headgruop bead of DDAB; 2D density maps of headgroup of (b) 50 wt%, (d) 60 wt% and (f) 70 wt% DDAB/water binary systems.

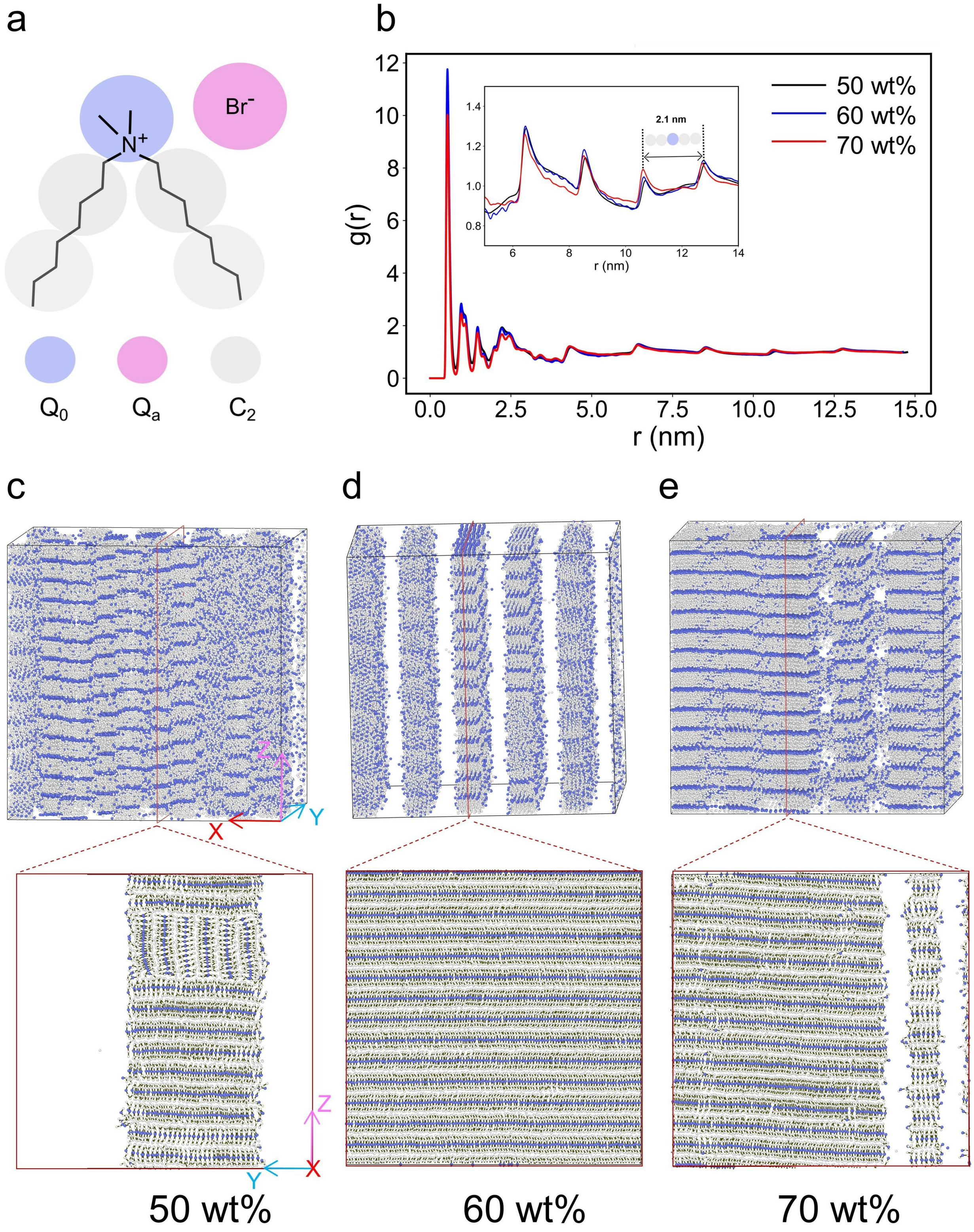

Figure S9. (a) Coarse-grained mapping of DOAB molecule; (b) radial distribution functions between headgroups of DOAB; snapshots of (c) 50 wt%, (d) 60 wt% and (e) 70 wt% DOAB/water binary systems.

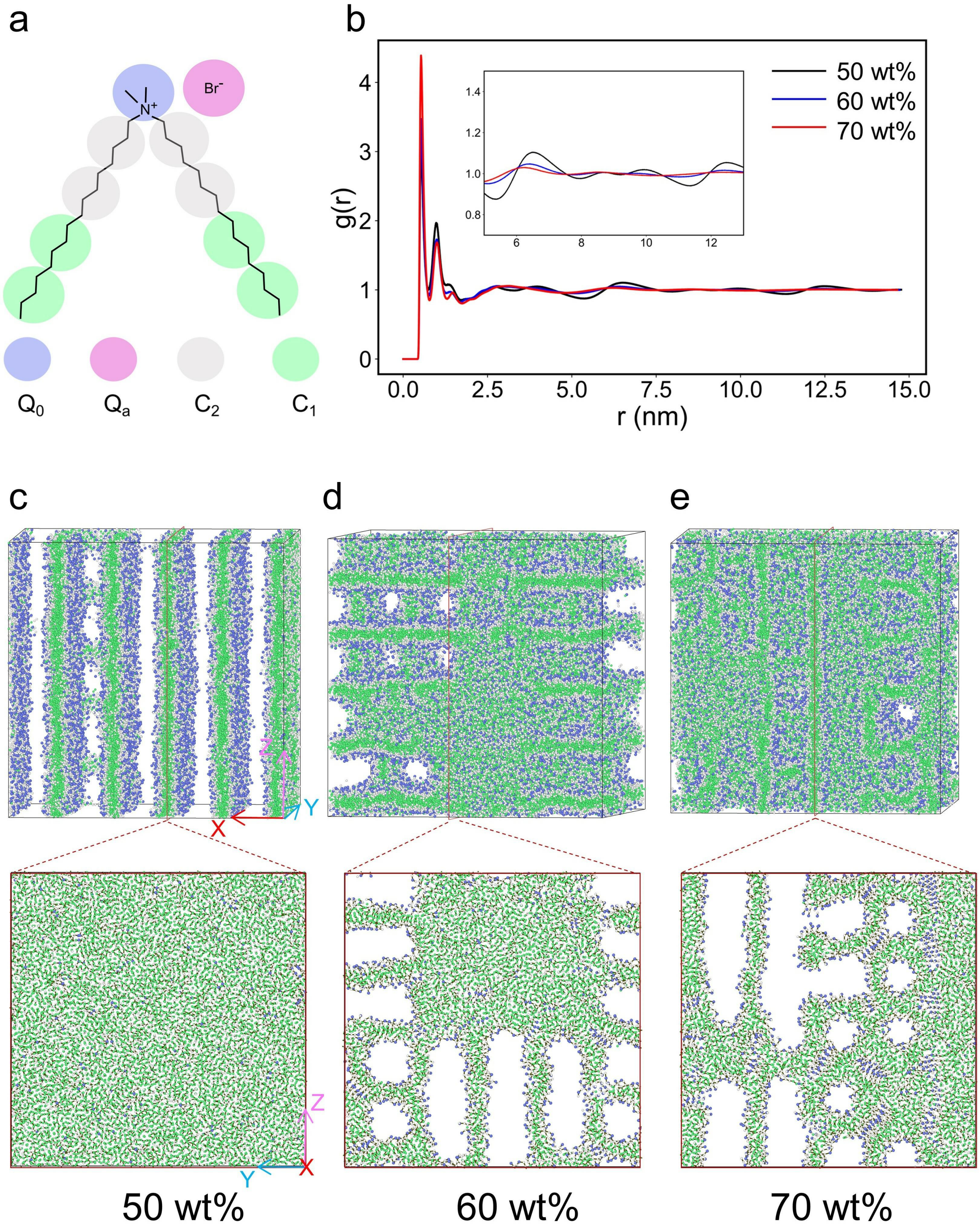

Figure S10. (a) Coarse-grained mapping of DPAB molecule; (b) radial distribution functions between headgroups of DPAB; snapshots of (c) 50 wt%, (d) 60 wt% and (e) 70 wt% DPAB/water binary systems.

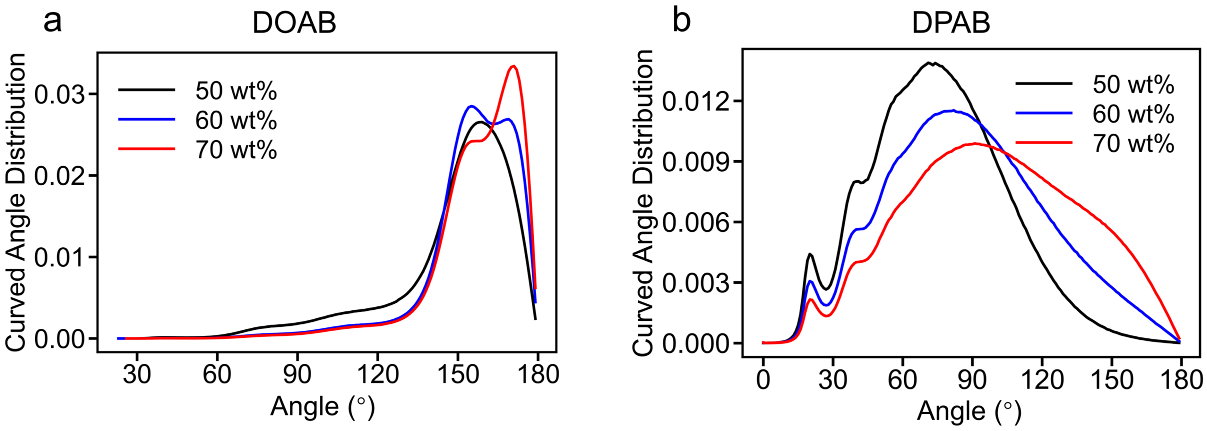

Figure S11. The curved angle distributions of (a) DOAB and (b) DPAB.

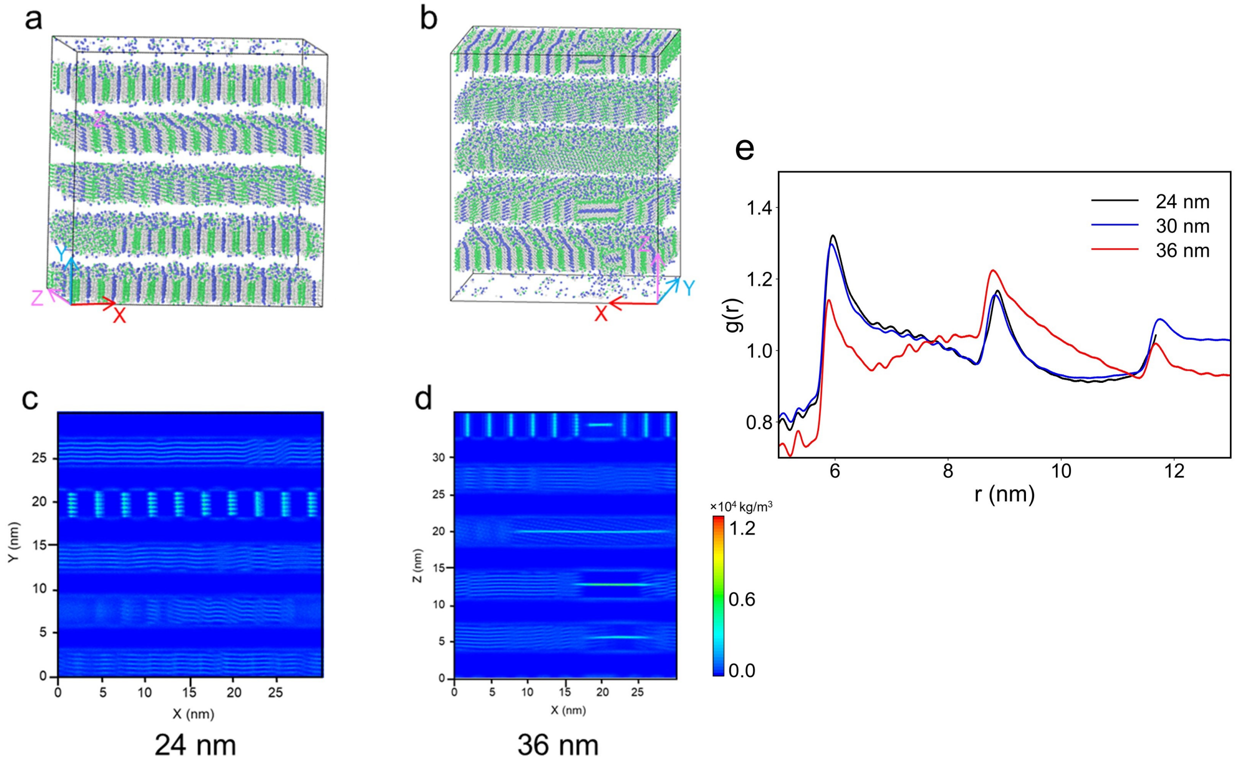

Figure S12. (a, b) Snapshots and (c, d) 2D density maps of headgroup of 60 wt% DDAB/water binary system using box sizes of (a, c) 24 nm and (b, d) 36 nm (Z dimension); (e) RDF profiles between headgroups of DDAB. As depicted in the provided image, even with these different box sizes, DDAB forms lamellar-in-lamellar structures in both cases with box sizes of 24 nm and 36 nm, indicating robust hierarchical organization. The RDF analysis further confirms the presence of this hierarchical structure with a well-defined long-range ordered arrangement.

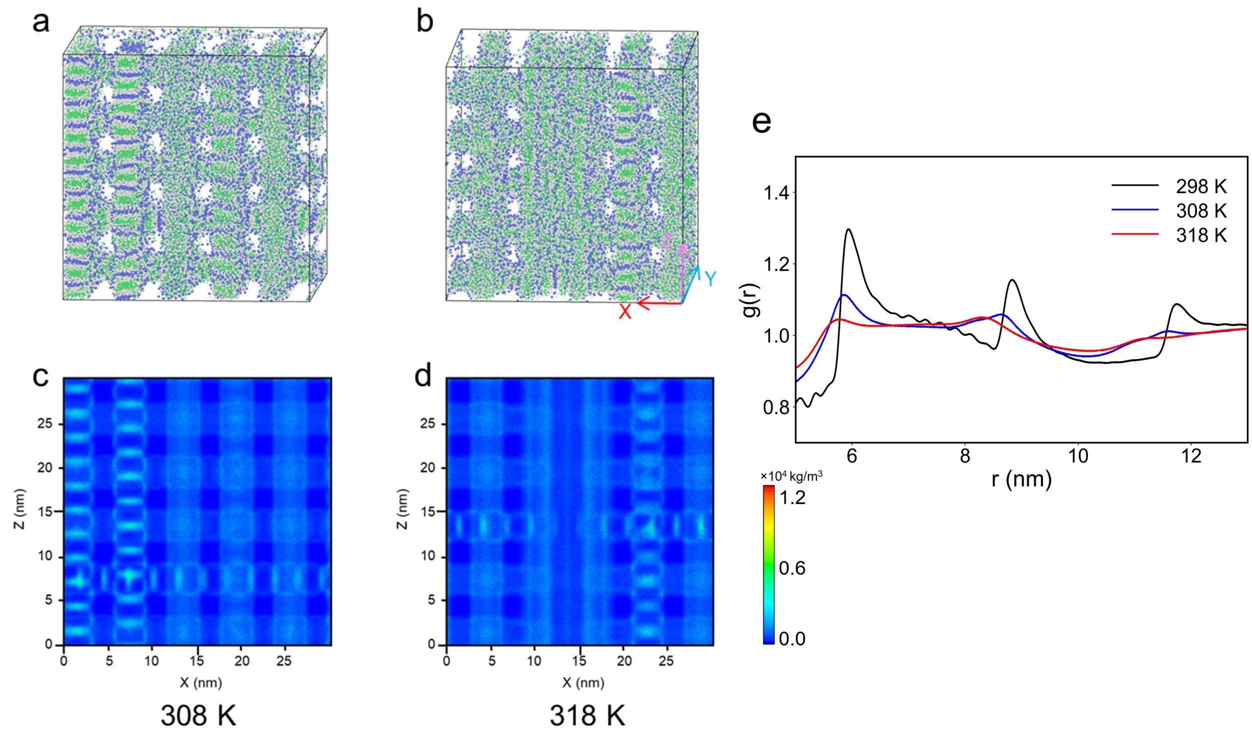

Figure S13. (a, b) Snapshots and (c, d) 2D density maps of headgroup of 60 wt% DDAB/water binary system at temperatures of (a, c) 308 K and (b, d) 318 K; (e) RDF profiles between headgroups of DDAB. These indicate that at higher temperatures, the stability of the lamellar-in-lamellar structure is compromised. Specifically, we observed that as the temperature increases, the outer lamellae (L=) tend to come into closer contact and undergo fusion. This phenomenon can be attributed to the increased thermal energy in the system with higher temperature, leading to enhanced molecular mobility and the merging of adjacent lamellae. Additionally, the well-defined long-range ordered arrangement is weakening and the molecular conformation of DDAB becomes more disordered under higher temperatures.

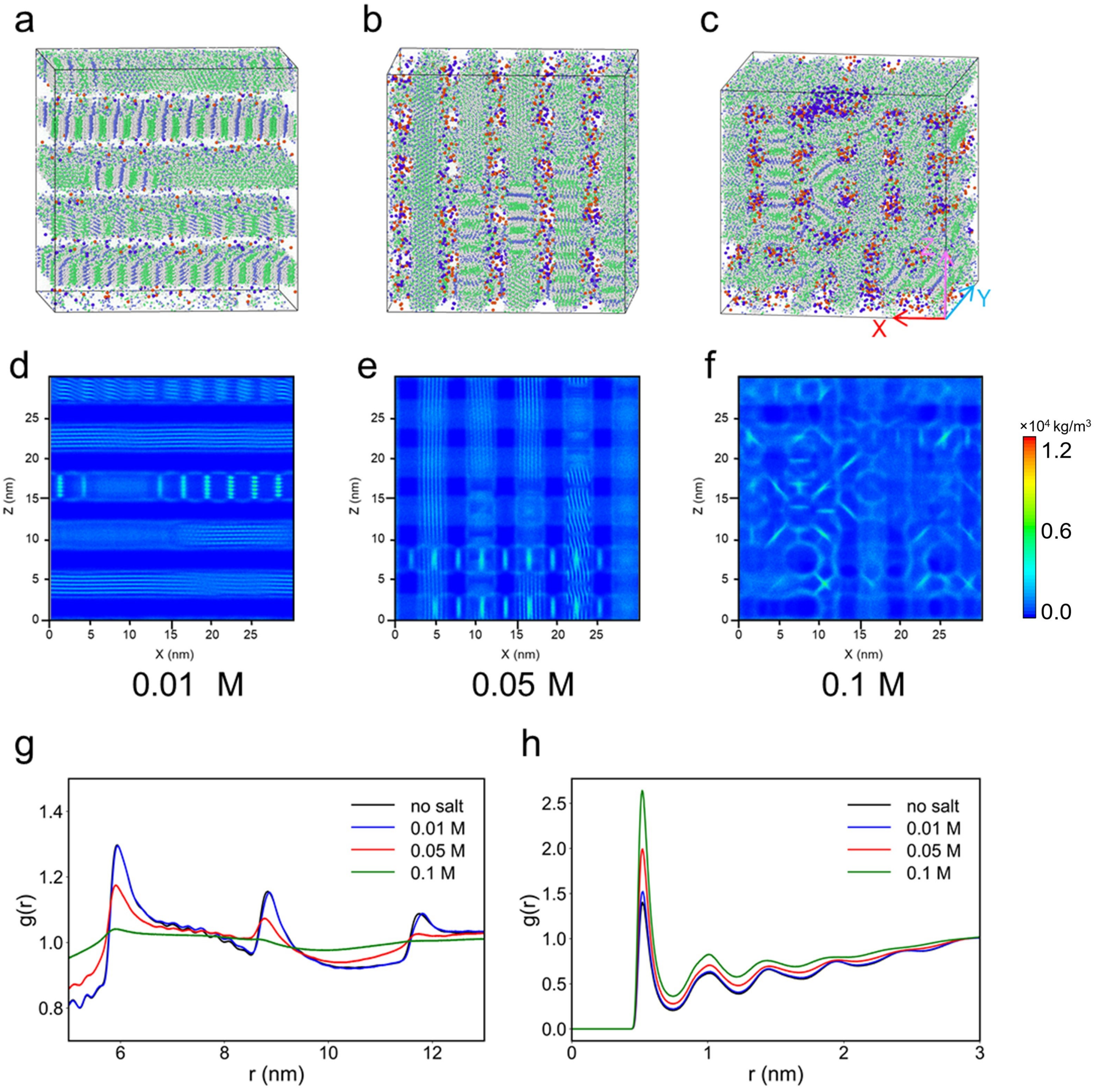

Figure S14. (a–c) Snapshots and (d–f) 2D density maps of headgroup of 60 wt% DDAB/water binary system at different NaCl concentrations of (a, d) 0.01 M, (b, e) 0.05 M, and (c, f) 0.1 M; RDF profiles of (g) headgroup to headgroup and (h) headgroup to water. Analogous to the impact of temperature, our results revealed that higher salt concentrations detrimentally affect the stability of the lamellar-in-lamellar structure, as evidenced by both qualitative observations in shapnots, density maps and quantitative analyses through RDF profiles.

References

(1) Marrink, S. J.; Risselada, H. J.; Yefimov, S.; Tieleman, D. P.; de Vries, A. H. The MARTINI Force Field: Coarse Grained Model for Biomolecular Simulations. *J. Phys. Chem. B* **2007**, *111*, 7812–7824.

(2) Yesylevskyy, S. O.; Schäfer, L. V.; Sengupta, D.; Marrink, S. J. Polarizable Water Model for the Coarse-Grained MARTINI Force Field. *PLoS Comput. Biol.* **2010**, *6*, e1000810.
